# A physician led approach to telehealth-enabled care coordination: innovation in reimbursement and delivery system models to support physician engagement

**Published:** 2012-06-15

**Authors:** Ileana Welte

**Affiliations:** Robert Bosch Healthcare, USA

**Keywords:** telehealth, physician based, reimbursement, quality, cost reductions, mortality reduction

## Abstract

**Introduction:**

The U.S. study, entitled, ‘Integrated Telehealth and Care Management Program for Medicare Beneficiaries with Chronic Disease Linked to Savings’ (Health Affairs, September 2011), explored the economic impact of using content-based remote patient monitoring combined with physician led patient-centred care management for high cost patients with congestive heart failure, chronic obstructive pulmonary disease and/or diabetes mellitus. Researchers conducted an analysis evaluating changes in healthcare spending resulting from physician led patient-centred care management supported by remote patient monitoring (n=1767) and demonstrated spending reductions of 7.7–13.3% (£197.17–£342.52) per intervention patient in acute hospital cost per quarter over the two-year period studied. In addition, significant mortality differences between the treatment and control groups were noted, which suggest that the intervention may have produced noticeable Improvements/changes in health outcomes.

**Aim and objectives:**

the presentation will outline the:

1. Findings from the analysis of the two-year study that could guide the design of future innovative physician led telehealth-enabled care coordination projects in the UK.
2. Parallels between the proposed UK local health system model as described by the Health and social care bill and the Medicare system in the US in the context of an innovative community-based physician led telehealth enabled integrated system of care.
3. Design and deployment of a system of care utilizing content-based telehealth technology to enable physician led integrated care coordination.

**Results:**

In an intent-to-treat population of 1725 patients, a reduction in critical aspects in utilization including hospitalization and A and E resulted in improved quality, satisfaction and cost outcomes for the patients and providers in the local region.

**Conclusion:**

Innovative reimbursement models drove physician led telehealth enabled care coordination that generated reductions in key utilization and cost while optimizing health status. The intervention also generated substantive efficiency improvements that led to substantive cost savings.

